# Dual-cytokine-armed oncolytic HSV for enhanced tumor immunotherapy

**DOI:** 10.1016/j.omton.2025.201082

**Published:** 2025-11-10

**Authors:** Ravi Dhital, Marcelo S.F. Pereira

**Affiliations:** 1 Center for Childhood Cancer Research, The Abigail Wexner Research Institute at Nationwide Children’s Hospital, Columbus, OH, USA

## Main text

### The evolving landscape of oncolytic virotherapy

Oncolytic viruses (OVs) have matured from a provocative concept into an established therapeutic modality capable of both direct tumor cytolysis and immune activation.^1^ Among viral backbones, herpes simplex virus type I (HSV-I) is a favored platform for oncolytic virus engineering, given its large genome, broad cellular tropism, and clinical precedence with talimogene laherparepvec (T-VEC), the first oncolytic virus approved by the Food and Drug Administration (FDA) to treat metastatic melanoma.^2^ These advances have reframed OVs not merely as lytic agents but as vectors for precise immunological engineering.

The next frontier for this promising therapy is to harness OVs as immune orchestrators, capable of delivering immunostimulatory cytokines, chemokines, or checkpoint modulators into the tumor microenvironment (TME) while preserving replicative capacity.^3^ Interleukin-12 (IL-12) and IL-15 are synergistic cytokines and are attractive transgenes for such approaches.^4^ Where IL-12 amplifies dendritic cell priming, promotes Th1 polarization, and enhances CD8^+^ effector activity, IL-15 maintains natural killer (NK) and memory CD8^+^ T cell survival.^5^ Their functional complementarity suggests the potential to promote an antitumor response when co-delivered.

In this issue of *Molecular Therapy Oncology*, Zhang and colleagues report the design of a dual-cytokine HSV-1 encoding both IL-12 and IL-15.^6^ Their findings demonstrate that simultaneous cytokine delivery enhanced infiltration of CD4^+^ and CD8^+^ T cells, as well as NK cells, into TME without compromising viral replication. These findings highlight the therapeutic potential of integrating synergistic cytokines into viral vectors, while raising concerns about their persistence, specificity, and translational safety.

### Engineering the virus: Replication and cytokine expression

The reported dual-cytokine oncolytic HSV (oHSV) was generated by deleting the α47 gene (encoding ICP47, an inhibitor of antigen presentation) and the inverted repeat region, while inserting IL-12 and IL-15 as transgenes. Replication kinetics in permissive Vero cells remained comparable to wild-type HSV-1, indicating that dual payload insertion did not erode viral competence. However, oHSV replication is generally less efficient in murine cells than in human cells, a limitation that attenuates the recapitulation of human viral replication in mouse disease models.^7^^,^^8^ Reflecting this, replication across murine tumor cell lines varied, with CT26 colon carcinoma cells supporting higher titers than B16F10 melanoma. This variation underscores the influence of tumor-intrinsic permissiveness on viral productivity, which may affect therapeutic efficacy across tumor types. Such variability highlights the need for systematic assessment of replication dynamics in clinically relevant models.

### Cytokine production and immune remodeling

The engineered oHSV expressed IL-12 and IL-15 in virus-permissive 293T cells *in vitro*, with cytokine secretion detectable at 16 h and peaking by 48 h post-infection. *In vivo*, intratumoral delivery in both models CT26 and B16F10 increased local IL-12 and IL-15 by day 6, accompanied by elevated interferon gamma (IFN-γ) and IL-4. These cytokine profiles correlated with increased intratumoral infiltration of CD4^+^ and CD8^+^ T cells and NKp46^+^ NK cells. Parallel expansion of these lymphocyte populations in the spleen, along with systemic secretion of IL-12 and IL-15 and reduced contralateral tumor burden, demonstrated that the therapy elicited a systemic antitumor immune response extending beyond the local tumor microenvironment.

Prior studies with single-cytokine-expressing oHSVs provide mechanistic context. IL-12-armed oHSV enhanced dendritic cell and macrophage activity and generated polyfunctional T cell responses,^9^ while IL-15-expressing oHSV increased NK cell cytotoxicity and supported memory CD8^+^ T cell persistence.^10^ The dual-cytokine construct reported by Zhang et al. integrated these functions into a single viral backbone. Their data suggest not only increased immune infiltration but also the potential for functional reprogramming of these immune subsets. However, infiltration alone does not equate to effective tumor control. Direct assessment of effector quality, including cytotoxic granule release, cytokine polyfunctionality, and exhaustion marker expression, was not performed and remains essential for defining therapeutic benefit.

### Significance and open questions

The study presented by Zhang et al. provides proof of concept that an oHSV can deliver multiple cytokines without compromising viral replication, achieving significant intratumoral immune cell infiltration. Despite the demonstration of T and NK cell expansion within the tumor, several critical uncertainties remain unexplored, such as the functional quality of these infiltrating lymphocytes. The antigenic specificity of the infiltrating lymphocytes is unresolved, as virus-specific responses may predominate, which could limit sustained tumor clearance once viral replication declines. Durability of immune infiltration also represents a key concern, as oHSV persistence in murine hosts is limited, and the long-term maintenance of immune infiltration and memory responses after viral clearance has not been established. Though the species origin of the transgenes is not specified, it should be presumed that they are murine and that a humanized version would need to be separately developed and validated for translation. Finally, although local expression of IL-12 and IL-15 is expected to mitigate systemic toxicity, chronic exposure of these cytokines may still drive excessive inflammation, immune exhaustion, or senescence, necessitating careful safety evaluation in immune-competent models.^11^ Addressing these questions will be essential to define whether dual cytokine oHSVs can progress beyond preclinical feasibility.

### Translational perspectives

The integration of IL-12 and IL-15 into an oHSV illustrates the next generation of “multifunctional” OVs. Future iterations may incorporate T cell engagers, checkpoint blockade, costimulatory ligands, or tumor-associated antigens to further enhance immune activation. Engineering strategies enabling tumor-restricted or inducible cytokine expression would mitigate risks of off-target inflammation. Advances in synthetic promoter design, microRNA targeting, and conditionally replicative backbones can refine cytokine expression profiles. Clinical translation will require testing in immune-competent antigenically relevant models. Rigorous evaluation in systems that recapitulate antigen heterogeneity, stromal architecture, and chronic inflammation is necessary to evaluate translational potential.

### Conclusion

This study demonstrates that dual-cytokine-armed oHSV can maintain replicative competence while inducing local IL-12 and IL-15 production, enhancing immune infiltration in tumors. Although unresolved issues remain regarding effector function, antigen specificity, durability, and safety, the findings highlight the promise of dual-cytokine-armed OVs as a strategy to enhance antitumor immunity. The work sets the stage for further exploration of combinatorial cytokines in virotherapy and represents a step toward expanding the therapeutic reach of OVs in cancer immunotherapy.

## Acknowledgments

We thank Clarity in Science Editing and Writing (https://clarityinscience.com) for English language scientific editing.

## Declaration of interests

The authors declare no competing interests.
